# Association of Very Rare *NOTCH2* Variants with Clinical Features of Alagille Syndrome

**DOI:** 10.3390/genes15081034

**Published:** 2024-08-06

**Authors:** Martina Ferrandino, Giovanna Cardiero, Fabiola Di Dato, Ylenia Cerrato, Luigi Vitagliano, Claudia Mandato, Filomena Morisco, Maria Immacolata Spagnuolo, Raffaele Iorio, Maria Donata Di Taranto, Giuliana Fortunato

**Affiliations:** 1 Dipartimento di Medicina Molecolare e Biotecnologie Mediche, Università degli Studi di Napoli Federico II, CEINGE-Biotecnologie Avanzate Franco Salvatore, 80131 Naples, Italy; 2 Dipartimento di Scienze Mediche Traslazionali, Università degli Studi di Napoli Federico II, 80131 Naples, Italy; 3 Istituto di Biostrutture e Bioimmagini, CNR, 80145 Naples, Italy; 4 Dipartimento di Medicina, Chirurgia e Odontoiatria “Scuola Medica Salernitana”, 84081 Baronissi, Italy; 5 Dipartimento di Medicina Clinica e Chirurgia, Università degli Studi di Napoli Federico II, 80131 Naples, Italy

**Keywords:** Alagille syndrome, cholestasis, genetics, *NOTCH2*, *JAG1*, pathogenicity evaluation, uncertain significance variants, missense variants, phenotype variability, bioinformatics

## Abstract

Background. Alagille syndrome (ALGS) is a rare autosomal dominant genetic disease caused by pathogenic variants in two genes: Jagged Canonical Notch Ligand 1 (*JAG1*) and Notch Receptor 2 (*NOTCH2*). It is characterized by phenotypic variability and incomplete penetrance with multiorgan clinical signs. Methods. Using Next Generation Sequencing (NGS), we analyzed a panel of liver-disease-related genes in a population of 230 patients with cholestasis and hepatopathies. For the rare variants, bioinformatics predictions and pathogenicity classification were performed. Results. We identified eleven rare *NOTCH2* variants in 10 patients, two variants being present in the same patient. Ten variants had never been described before in the literature. It was possible to classify only two null variants as pathogenic, whereas the most of variants were missense (8 out of 11) and were classified as uncertain significance variants (USVs). Among patients with ALGS suspicion, two carried null variants, two carried variants predicted to be pathogenic by bioinformatics, one carried a synonymous variant and variants in glycosylation-related genes, and two carried variants predicted as benign in the PEST domain. Conclusions. Our results increased the knowledge about *NOTCH2* variants and the related phenotype, allowing us to improve the genetic diagnosis of ALGS.

## 1. Introduction

Alagille syndrome (ALGS) is a rare genetic disease with autosomal dominant transmission, mainly caused by pathogenic variants in two gene-encoding proteins involved in the Notch Signaling Pathway: Jagged Canonical Notch Ligand 1 (*JAG1*) and Notch Receptor 2 (*NOTCH2*) [1]. 

Both JAG1 and NOTCH2 are transmembrane proteins acting in signal transduction, being the first the ligand expressed on signaling sending cells and the second the receptor expressed on signaling receiving cells. When JAG1 binds to NOTCH2, the latter can be cleaved, releasing the intracellular portion in the cytoplasm, where it can interplay with other signaling pathways, or it can be translocated in the nucleus participating in the transcription activation of target genes. NOTCH2 signaling is essential in the cell differentiation during embryogenesis and particularly in the differentiation of bile duct epithelial cells [2]. Then, a defective interaction of JAG1 and NOTCH2 can show several morphological alterations in the body, including the bile duct paucity as a main consequence.

ALGS was first described as an arteriohepatic dysplasia [3]. It is now considered a disease with multiorgan symptoms with incomplete penetrance, leading to variable phenotypes that could hamper the clinical diagnosis. The main feature of ALGS is the bile duct paucity revealed by liver biopsy and causing cholestasis with the associated increase in γ-glutamyl transferase (GGT), and, sometimes, itch, xanthomas, and cirrhosis. The most serious consequence of ALGS is the need for liver transplantation, which in the more severe cases can be necessary in the first year of life.

To date, the clinical diagnosis is based on the presence of 3 out of 7 of the following signs: cholestasis, congenital cardiac defects, ophthalmologic abnormalities (mainly posterior embryotoxon), skeletal malformation (mainly butterfly vertebrae), typical dysmorphic facies, renal abnormalities (mainly renal dysplasia), and vascular alterations [4,5,6].

The genetic diagnosis based on the presence of pathogenic variants in *JAG1* and *NOTCH2* genes could support the ALGS in case of atypical manifestations [7]. In particular, about one third of patients presenting with less than 3 clinical features showed variants in *JAG1* [8].

The aim of our study is to report rare *NOTCH2* variants along with the clinical phenotype of the carrying patients to increase the knowledge about the clinical implications of *NOTCH2* variants on the ALGS signs. This approach could also be helpful to better define the variant pathogenicity, according to the criteria of American College of Medical Genetics and Genomics (ACMG) [9], which consider the identification of the variant in several unrelated patients with clinical features of the disease as a support to the variant pathogenicity.

## 2. Materials and Methods

### 2.1. Patients

The starting cohort consisted of 230 white patients with cholestasis and hepatopathies who were analyzed by Next Generation Sequencing (NGS) for a panel of 59 genes associated with liver diseases. Patients were recruited at the pediatric liver clinic of the Dipartimento di Scienze Mediche Traslazionali of Università degli Studi di Napoli Federico II (Naples, Italy), at the adult liver clinic of Dipartimento di Medicina Clinica e Chirurgia of Università degli Studi di Napoli Federico II (Naples, Italy), and at the pediatric liver clinic of the Azienda Ospedaliera Universitaria San Giovanni di Dio e Ruggi d’Aragona (Salerno, Italy). Written informed consent was obtained for each patient. The study was performed in accordance with the Declaration of Helsinki and was approved by the Ethical Committee of the Università degli Studi di Napoli Federico II (Number 77/21, 26 March 2021).

### 2.2. Genetic Analysis

Genomic DNA of each patient was extracted from peripheral blood using ReliaPrep™ Blood gDNA Miniprep System, according to the manufacturer’s instructions (Promega, Madison, WI, USA). All patients were analyzed for a panel of 59 genes associated with hepatopathy and cholestasis, including the genes associated with ALGS (*JAG1* and *NOTCH2*). For each gene, all the exons, the flanking intronic regions (±25), the 3′UTR, and the 5′UTR were analyzed. Copy number variants were not evaluated by this approach.

The libraries were obtained using a SureSelect QXT Target Enrichment kit for Illumina Sequencing (Agilent Technologies, Santa Clara, CA, USA). High-throughput sequencing was performed on an Illumina MiSeq platform (llumina Inc., SanDiego, CA, USA), and then data analysis was conducted using Seqr (Broad Institute). 

The variant positions were reported according to the Human Genome Variation Society nomenclature using the following reference sequences: *NOTCH2* NM_024408.4; *JAG1* NM_000214.3; *COG5* NM_006348.5; *ALG1* NM_019109.5. GnomAD v4.1.0 database (https://gnomad.broadinstitute.org/ access date 18 June 2024) was used to establish the minor allele frequency (MAF) of the variant (access date 18 June 2024), being the widest available population database to date. An HGMD professional version 2024.1 and ClinVar (https://www.ncbi.nlm.nih.gov/clinvar/ access date 18 June 2024) were used to verify previous reports of the genetic variants, together with the associated published papers (access date 18 June 2024).

The variant pathogenicity was evaluated according to the ACMG guidelines [9]. 

### 2.3. Bioinformatic Predictions

Several tools were used to predict the functional effect of the identified variants. For the missense variants, the following tools were used to predict the pathogenicity due to the amino acid substitution: MutationTaster2021 (https://www.mutationtaster.org/ access date 18 June 2024), PolyPhen-2 Hvar (http://genetics.bwh.harvard.edu/pph2/ access date 18 June 2024), SIFT (https://sift.bii.a-star.edu.sg/www/SIFT_aligned_seqs_submit.html access date 18 June 2024), Align GVGD (http://agvgd.hci.utah.edu/links.php access date 18 June 2024), REVEL (https://genome.ucsc.edu/cgi-bin/hgTrackUi?db=hg19&g=revel access date 18 June 2024), CADD (version 1.6) (https://cadd.gs.washington.edu/ access date 18 June 2024), and alpha-missense (https://github.com/google-deepmind/alphamissense access date 18 June 2024). The prediction of splicing alterations was performed for all non-null variants (missense, synonymous, and non-canonical splice sites) by the following tools: SpliceSiteFinder 3.1, MaxEntScan, NNSPLICE (version 0.9), and GeneSplicer accessed by Alamut Visual Plus version 1.8 (Sophia genetics). Alamut Visual Plus was also used to evaluate alterations in the predicted branch sites. Splicing alterations were reported if the variant was predicted (1) to alter the canonical splicing by more than 50% of score; (2) to induce an increase/decrease in the prediction scores relative to cryptic splice sites or branch points by more than 50% of the score. The abolition of branch points in exonic regions was not considered because these are not physiological positions for this type of splicing-related sequences.

## 3. Results

### 3.1. Identification of Rare Variants in NOTCH2

Eleven rare variants of *NOTCH2* were identified (Table 1), among which only one, the nonsense variant c.6007C>T–p.(Arg2003*), was previously identified in patients with ALGS [10,11,12,13]. 

Considering an estimated prevalence of the ALGS ranging from 1:30,000 to 1:70,000 [7,14], a maximum MAF of 0.003% should be considered for potential association with this disease. This condition was satisfied by all variants but the synonymous one, i.e., the c.5103A>G, p.(Lys1701=), which showed a MAF of 0.03% both in the total population and in the European non-Finnish sub-group, to which belong the analyzed patients. Three variants were not present in GnomAD, indicating that they are so rare as to be absent in more than 1.6 million alleles, i.e., more than 800,000 subjects. The other variants showed a MAF ranging from 0.0006% to 0.0012%, being then compatible with the ALGS prevalence. None of the variants were identified at the homozygous status in GnomAD.

Two null variants were identified, a deletion of eight bases causing frameshift (never reported and absent in GnomAD) and the nonsense variant in the exon 33. Both these null variants were classified as pathogenic for the dramatic impact on the protein (criterion PVS1 according to [9]). Aside from the above synonymous variant, the other eight variants were missense in exons 4, 18, 19, 25, 27, 33, and 34. The variants p.(Val1623Gly) and p.(Asp2004Val) were identified in the same patient, but the unavailability of parent genetic data make it impossible to establish if they are present in the same allele or in different ones.

### 3.2. Bioinformatic Predictions of Variants Role and Pathogenicity Classification

For the missense variants, we performed the evaluation of the impact of the amino acid substitution by six individual prediction tools and by two tools based on multiple evidence (REVEL and MetaRNN). A pathogenicity prediction according to ≥7/8 tools was present for three variants: c.665A>G–p.(Tyr222Cys); c.4868T>G–p.(Val1623Gly); c.6011A>T–p.(Asp2004Val), being the last 2 variants present in the same patient (Table 2).

According to these predictions, four variants [p.(Gln921Arg), p.(Glu1488Lys), p.(Ser2142Pro), and p.(Phe2268Leu)] could be considered not impacting the protein function, being predicted as pathogenic by ≤ 2 tools, whereas the variant p.(Arg1048Cys) reported ambiguous results, being predicted as pathogenic by 4/8 tools (Table 3). The last variant created a cysteine residue in the EGF-like domain, a region in which the disulfide bounds between two cysteines is essential for the protein folding. Taken together, among the eight missense variants, two variants led to the creation of a cysteine in the EGF-like domain, which is a cysteines-rich region where folding is strictly regulated by disulfide bonds. 

Since the variant effect could also be due to a change in the mRNA sequence leading to an altered splicing, we performed the prediction of alterations relative to splice sites and branch point consensus sequences for all the missense variants and for the synonymous variant (Table 3). None of the variants were predicted to induce a decreased utilization of the canonical splice sites. The variants c.2762A>G–p.(Gln921Arg) and c.6802T>C–p.(Phe2268Leu) were predicted to increase the utilization of a cryptic splice site by 1/4 and 2/4 prediction tools, respectively. However, the total absence of a predicted splice site consensus by the other tools makes these predictions not completely reliable. Both variants belong to the group of variants for which an impact of the amino acid substitution could be confidently excluded by predictions. 

The alteration prediction of branch point consensus sequences was also performed (last column of Table 3) indicating a potential creation of a branch point with a modest score (62.7/100) for the variant c.4462G>A–p.(Glu1488Lys) that was predicted not impacting the protein sequence. The abolition of the predicted branch point at c.6011 by the variant c.6011A>T–p.(Asp2004Val) was not considered because this type of sequences is not expected to be in exons. 

In conclusion, three missense variants (two of which present in the same patient) were predicted to impact the protein structure/function, whereas the splice site and branch point analysis did not reveal any strong prediction of pathogenicity.

### 3.3. Analysis of ALGS-Related Clinical Signs 

The demographic and clinical characteristics of the 10 patients carrying *NOTCH2* variants are reported in Table 4. This table is focused on the clinical features typically observed in ALGS. Seven out of ten patients (patients 1, 2, 6, 7, 8, 9, and 10) had a clear suspicion of ALGS, showing at least three of its typical clinical signs. Cholestasis was present in all these patients. These patients include all the patients with a clinical onset before 3 months of age and a child of 4 years. The remaining three patients showed at least one clinical feature of ALG, including hepatic alterations but not cholestasis. These patients include two children of 4 and 5 years and an adult man with a metabolic syndrome. 

All variants identified in the three patients without a clear ALGS suspicion are the missense variants with poor pathogenicity predictions. The p.(Gln921Arg) variant was identified in the adult man with metabolic syndrome (type II diabetes; arterial hypertension; hyperuricemia; mixed dyslipidemia), liver steatosis, and a renal cyst (patient 3). The p.(Arg1048Cys) variant was identified in the 5-year-old, patient 4, with a mild ALGS facies and isolated hypertransaminasemia. Despite the fact that this variant was predicted as impacting the protein function by a few prediction tools, it introduced a cysteine residue that could alter the disulfide bond pattern and then the protein structure. The p.(Glu1488Lys) variant was identified in a 3-year-old patient with only biliary sludge and cardiac alterations (patient 5). 

As to the patients with a clear ALGS phenotype carrying only a variant in *NOTCH2*, patient 1 with a non-neonatal-onset (4 years) carried the variant p.(Tyr222Cys), which introduced a cysteine and was predicted as pathogenic by missense tools. Patient 8 carried the pathogenic nonsense variant c.6007C>T–p.(Arg2003*), clearly confirming the genetic alteration inducing the ALGS suspicion. Patient 10 carried the missense variant c.6802T>C–p.(Phe2268Leu) in the exon 34, encoding for the PEST domain. The variant only showed an ambiguous prediction of splicing alterations. However, the patient only showed a moderate expression of ALGS-related signs.

In patient 6, who had a clear ALGS clinical suspicion, it was not possible to define if the two *NOTCH2* variants were present on the same allele (heterozygous patient with an encoded protein carrying two amino acid substitution) or on the two different alleles making the patient a compound heterozygote. Due to the lethality of the bi-allelic pathogenic variants in *NOTCH2*, we hypothesized that the two variants were on the same allele or if the two variants were present on different alleles, only one was pathogenic. However, both variants are so rare as to be absent in GnomAD and were predicted as pathogenic by an in silico analysis. 

Table 4 also reports the rare variants in other genes identified in these patients. No patient showed variants in genes related with other genetic cholestatic diseases. 

Patient 2 carried both a deletion with frameshift in *NOTCH2* c.1583_1590delTTTGCCAG–p.(Val528Aspfs*2), classified as pathogenic and a rare missense variant in *JAG1*: (c.94T>C–p.(Ser32Pro). The last variant is a so rare that it was absent in the large population database GnomAD (PM2 according to [9]). Furthermore, this variant was not reported in HGMD or in ClinVar. The variant was predicted as pathogenic by 6/8 tools for protein function (PolyPhen Hvar, SIFT, CADD, alpha-missense, REVEL, and Meta RNN) and was predicted to create a new potential splice site at c.97 (score 0.49/1) by a single splicing tool, NNSPLICE. Due to the predominant evaluation of pathogenicity for the impact on protein function by the amino acid substitution, a supporting criterion could be added (PP3 according to [9]). The final classification of the *JAG1* variant was USV. The patient phenotype can be confidently attributed to the frameshift variant in *NOTCH2*, but it cannot be excluded that the *JAG1* USV could act to worsen the phenotype. In fact, this was the only patient undergoing liver transplantation among the described population. 

Patient 7 showed a clinical presentation compatible with ALGS, although the synonymous variant c.5103A>G–p.(Lys1701=) showed a frequency too high for the disease prevalence. According to the in silico prediction, the variant did not induce splicing alterations. Patient 7 also carried heterozygous variants in *ALG1* and *COG5* genes causative of the autosomal recessive disorders: congenital disorder of glycosylation (CDG), type Ik; and CDG, type III, respectively. The variant c.946G>A–p.(Val316Ile) in *ALG1* showed a MAF of 0.03% in GnomAD, with the presence of four homozygotes among more than 800,000 subjects. A ClinVar submission reported that this variant was identified in a patient with *ALG1*–CGD at the compound heterozygous status. The *COG5* variant c.298C>T–p.(Leu100Phe) showed a MAF of 0.08% in GnomAD, with the presence of 2 homozygotes. A patient with CDG was reported in ClinVar as having this variant at compound heterozygosis with a likely benign variant. Since the variant is present in a functional domain, a moderate pathogenicity criterion can be assigned. Based on the available data, both variants in *ALG1* and *COG5* were classified as USV. However, even if the variants will be classified as pathogenic in the future, the heterozygous status makes the patient not be affected by CDGs, whose clinical consequences (mainly neurologic and with hepatomegaly among liver-related features) were not present in the patient. The patient’s mother, without any clinical sign of ALGS, was genetically analyzed and was heterozygous for the *NOTCH2* c.5103A>G–p.(Lys1701=) and *ALG1* c.946G>A–p.(Val316Ile) variants. Since it is well known that the ALGS has an incomplete penetrance, we cannot confidently assign the benignity criterion about the lack of co-segregation to the *NOTCH2* variant. The additive effect of both variants in the CDG-related genes with a mild NOTCH2 impairment at the base of the phenotype could not be excluded.

Patient 9, carrying the c.6424T>C–p.(Ser2142Pro) variant, was also analyzed by the CGH array for psychomotor impairment and showed carriers of several chromosomal alterations of copy number variants, among which only the duplication could likely be considered pathogenic. Although the patient showed a clear ALGS phenotype since presenting cholestasis, the typical facies, cardiac, vascular and renal features, it cannot be excluded that these clinical signs could be partially due the chromosomal rearrangements.

## 4. Discussion

ALGS shows a very variable phenotype, including seven different clinical signs that are not usually present in all patients, making the clinical diagnosis very difficult. The presence of a pathogenic variant by the genetic screening helps the identification of the mildest cases, also avoiding the invasive liver biopsy need to detect the typical bile duct paucity.

Most of the described ALGS cases carried variants of *JAG1*, whereas only 2.5% of cases carried variants of *NOTCH2* [11]. The *NOTCH2* gene was identified more recently [15] as a gene causative of ALGS, and the reported, clearly pathogenic variants are still few, being only 28 (16 missense and 12 null variants) in HGMD professional. The lack of pathogenicity evidence, including the absence of previous reports, makes it difficult to assign a clear role to the rare *NOTCH2* variants. We identified 11 variants, among which only the nonsense variant c.6007C>T–p.(Arg2003*) was previously reported [10,11,12,13]. Most of the identified variants are missense (8/11), as previously described in other populations [10,11,13,16], suggesting a low tolerance for null *NOTCH2* variants. In our study, a pathogenic role could be established only for the two null variants, the previous reported nonsense c.6007C>T–p.(Arg2003*), and the novel deletion/frameshift c.1583_1590delTTTGCCAG–p.(Val528Aspfs*2). All other variants were classified as USV, i.e., variants that could have, as well could not have, a pathogenic role but that, with the current knowledge, cannot be confidently classified as pathogenic or benign.

Thanks to the NGS, wide genetic analyses including all genes associated with cholestasis are currently being performed in patients with hepatopathies, leading to the identification of several new very rare variants. When a *NOTCH2* variant is identified during the molecular analysis for cholestasis, only few pathogenic criteria could be considered, due to the scarcity of information about it. Accordingly, the most recent study that analyzed both variants of *NOTCH2* identified in a large Chinese population and those described in previous studies reported the difficulty of classifying missense variants, with only data about the variant frequency and the bioinformatic predictions often being available [13]. Bioinformatics should be considered as just prediction, and the incomplete sensitivity and specificity of the predictions of all available tools was demonstrated, making it necessary to perform the analysis with several different tools [17,18]. It should be noted that many *JAG1* and *NOTCH2* variants affect the protein function by altering the correct glycosylation [1,19], an aspect that is not fully evaluated by the used bioinformatic tools. This aspect implies that a variant predicted as benign could alter the correct glycosylation pattern, affecting the protein function.

Our report, together with all other reports about unclassified variants in *NOTCH2* gene, could be helpful to enlarge the knowledge of cholestasis-associated variants. In addition, our study design allows for defining the prevalence of *NOTCH2* variants among patients with different liver alterations and evaluating the different expressions of the ALGS disease due to *NOTCH2* variants. Also for *JAG1*, it was described that one third of patients with only one or two clinical signs of ALGS carried pathogenic variants in *JAG1* [8]. This aspect could indicate that the presence of variants in *JAG1*, as well as *NOTCH2*, could also induce mild phenotypes not clearly classifiable as ALGS. A possible explanation for this phenomenon could be the presence of modifier genes or genetic variants that were already hypothesized [20].

Genes involved in protein glycosylation have been proposed as modifier genes thanks to murine models [21,22], whereas in human data derived from a genome-wide association study the association of SNPs in the *THBS2* upstream region are revealed [23]. As for glycosylation-related genes, we identified a patient with a mild phenotype of ALGS carrying a not so rare synonymous variant (c.5103A>G, p.(Lys1701=), MAF = 0.03%) without predicted splicing alterations together with two variants at heterozygous status in two genes causative of CDG. Although the pathogenic mechanism was not clearly clarified by the in silico predictions, it could be hypothesized that this variant could be considered just an hylomorphic allele (not completely impairing the encoded protein), which, together with a double partial glycosylation defect, induces the ALGS phenotype development. To date, no data have been presented in the scientific literature about the possible role of these CDG-related genes in ALGS’ phenotypic expression and penetrance.

The variability of clinical manifestations could be partially related to the genetic causes. In fact, it was described that patients with *NOTCH2* pathogenic variants have lesser cardiac and vertebral symptoms than patients with *JAG1* pathogenic variants, and the presence of the typical facies is less frequent [10]. In our seven cases with a clinical suspicion of ALGS, we identify only two patients with butterfly vertebrae, but a consistent number of patients with cardiac alterations (5/7) and with the facies (4/7).

In our population, all patients carrying *NOTCH2* variants did not carry other variants leading to other genetic cholestasis. In the NGS era, the molecular screening should include all genes involved in genetic cholestasis, including those causing the different forms of progressive familial intrahepatic cholestasis (PFIC), the Dubin–Johnson syndrome, but also the poorly known cerebrotendinous xanthomatosis associated with neonatal cholestasis [24,25,26,27]. The extreme expression variability of genetic cholestasis, like other genetic diseases including cardiomyopathies or dyslipidemias, makes the clinical diagnosis very hard, requiring the support of a definite genetic identification of the molecular defect to correctly monitor or treat the patients [28,29]. The identification of the genetic alteration could also be useful to extend the diagnosis to other family members. Since this is a dominant disease, the genetic diagnosis of a patient indicates that one half of the progeny will suffer from ALGS. Prenatal molecular diagnosis could be helpful in this disease with very early manifestations, as well as in other genetic diseases [30,31]. A non-invasive approach was successfully applied to dominant diseases including ALGS [32].

It was reported that a quarter of ALGS-causative missense variants in *JAG1* alter the cysteine number by creating or eliminating a cysteine residue [11]. We identified 2/11 variants leading to a cysteine gain (2/8 of missense variants, i.e., a quarter of identified missense variants). According to a recent report, among 47 missense variants from the analyzed population and previous reports, 9 involve an alteration of cysteine number (5 loss and 4 gain of cysteine) [13]. The EGF-like domain is the NOTCH2 region that contains the most cysteines being folded by a high number of disulfide bonds. However, the whole EGF-like domain is a strictly conserved structure, and all the amino acid substitutions not involving a cysteine could affect the creation of near disulfide bonds.

The three patients without a clear ALGS suspicion carried missense variants with poor pathogenicity predictions. Among the seven patients with ALGS suspicion, 2 carried null variants, one of which was in double heterozygosis with a missense *JAG1* variant previously unreported. This patient was the most severe case of the population, being the only one undergoing liver transplantation. Despite a clear ALGS clinical suspicion of patient 6, who carried 2 very rare missense variants, it is not possible to establish which one of the identified variants is the responsible of the phenotype and even if the clinical presentation is the result of the contemporary presence of the two variants. To the best of our knowledge, patients with biallelic pathogenic variants were never described, whereas it was reported that homozygous mutant mice die during embryonic development or perinatally in cases of hypomorphic variants [33,34]. The other variant predicted to be pathogenic was a missense variant in the EGF-like domain introducing a cysteine–p.(Tyr222Cys).

The variants predicted to be benign are the synonymous one (in the patient with variants in the CDG-related genes) and the 2 missense variants in the exon 34–p.(Ser2142Pro) and p.(Phe2268Leu), encoding the PEST domain, a protein region involved in the protein degradation. Variants in this region could impair the protein degradation, causing a protein accumulation with consequent gain in function and leading to the Hajdu–Cheney syndrome [35]. However, recent reports suggested the presence of variants in this domain among ALGS patients [13,36]. One of our patients with a variant in the exon 34 also carried several chromosomal rearrangements detected by CGH array that could contribute to his complex phenotype.

The limitations of this study include the lack of clinical evaluation and genetic analysis of most patient parents and other relatives, making it impossible to perform the segregation analysis within families, as well as to establish the allelic disposition of two *NOTCH2* variants in a patient. Furthermore, since copy number variants were not analyzed, it cannot be excluded that some patients could carry a deletion duplication in cholestasis-associated genes.

## 5. Conclusions

With the development of NGS, the molecular analysis of many genes becomes possible, making the research of variants in *NOTCH2* in ALGS patients a standard procedure, although leading to many variants that cannot be clearly interpreted. In this study, we reported 11 variants of the *NOTCH2* gene, 10 of which were never described before in patients with cholestasis/hepatic diseases. A pathogenicity was established only for the null variants, whereas all the others were classified as USV. Describing the variants, alongside the clinical signs of patients in which they were identified, our results will increase the knowledge about *NOTCH2* variants and the related phenotype, being useful to refine the pathogenicity evaluation of *NOTCH2* variants. Finally, this paper highlights that a wide genetic screening could be helpful both at excluding the other genetic cholestasis and at evaluating glycosylation-related genes that are usually not investigated.

## Figures and Tables

**Table 1:** Characteristics and pathogenicity classification of identified *NOTCH2* variants.

Nucleotide	Protein	GnomAD MAF–Allele Count–Homo (Analyzed Allele Number)		Variant Type	ACMG Classification	Exon	Domain	SNP ID
		Total	European (Non-Finnish)					
c.665A>G	p.(Tyr222Cys)	0.0006%–10–0 (1,613,888)	0.0006%–7–0 (1,179,876)	Missense	USV	4	EGF-like	rs782566552
c.1583_1590 delTTTGCCAG	p.(Val528Aspfs*2)	NR	NR	Frameshift	Pathogenic	10	EGF-like	NR
c.2762A>G	p.(Gln921Arg)	0.0012%–19–0 (1,613,262)	0.0013%–16–0 (1,179,338)	Missense	USV	18	EGF-like	rs377647478
c.3142C>T	p.(Arg1048Cys)	0.0011%–18–0 (1,614,080)	0.0008%–9–0 (1,179,992)	Missense	USV	19	EGF-like	rs782077143
c.4462G>A	p.(Glu1488Lys)	0.0008%–13–0 (1,614,140)	0.0008%–10–0 (1,180,046)	Missense	USV	25	NRR	rs1131691315
c.4868T>G ^#^	p.(Val1623Gly)	NR	NR	Missense	USV	27	NRR	NR
c.5103A>G	p.(Lys1701=)	0.03%–482–0 (1,614,038)	0.03%–342–0 (1,180,008)	Synonymous	USV	28	Undefined	rs201233415
c.6007C>T	p.(Arg2003*)	0.00006%–1–0 (1,612,624)	absent (1,179,038)	Nonsense	Pathogenic	33	ANK5	rs312262801
c.6011A>T ^#^	p.(Asp2004Val)	NR	NR	Missense	USV	33	ANK5	NR
c.6424T>C	p.(Ser2142Pro)	0.0003%–5–0 (1,614,180)	0.0004%–5–0 (1,180,020)	Missense	USV	34	PEST	rs2101144199
c.6802T>C	p.(Phe2268Leu)	0.0006%–10–0 (1,614,130)	0.0004%–5–0 (1,180,026)	Missense	USV	34	PEST	rs149580724

^#^ variants identified in the same patient; MAF = minor allele frequency; NR = not reported; USV = variant of uncertain significance; EGF = epidermal growth factor; NRR = negative regulatory region; ANK5 = ankyrin repeats 5; PEST = proline (P), glutamic acid (E), serine (S), and threonine (T) degradation domain.

**Table 2:** Pathogenicity predictions of *NOTCH2* missense variants for their impact on protein function.

Nucleotide	Protein	Mutation Taster	PolyPhen Hvar	SIFT	PROVEAN	CADD	Alpha-Missense	REVEL	Meta RNN	Missense Pathogenicity Predicition *
c.665A>G	p.(Tyr222Cys)	B	PD	D	PD	PD	D	PD	PD	7/8
c.2762A>G	p.(Gln921Arg)	B	B	B	B	A	B	B	B	0/8
c.3142C>T	p.(Arg1048Cys)	B	PD	B	A	PD	A	PD	PD	4/8
c.4462G>A	p.(Glu1488Lys)	B	B	B	B	A	A	PD	A	1/8
c.4868T>G ^#^	p.(Val1623Gly)	D	PD	D	PD	PD	D	A	PD	7/8
c.6011A>T ^#^	p.(Asp2004Val)	D	PD	D	PD	PD	D	PD	PD	8/8
c.6424T>C	p.(Ser2142Pro)	B	B	B	B	A	B	B	B	0/8
c.6802T>C	p.(Phe2268Leu)	B	B	D	B	A	D	B	B	2/8

^#^ variants identified in the same patient; * number of tools predicting damaging effects; B = benign; D = deleterious; PD = probably or possibly damaging; A = ambiguous.

**Table 3:** Predictions of splicing alterations induced by the *NOTCH2* variants.

Nucleotide	Protein	SpliceSiteFinder (max 100)	Max EntScan (max 12)	NNSPLICE (max 1)	GeneSplicer (max 24)	Branch Points (max 100)
c.665A>G	p.(Tyr222Cys)	N	N	N	N	N
c.2762A>G	p.(Gln921Arg)	N	N	0 → 0.40 gain PSS at c.2753	N	N
c.3142C>T	p.(Arg1048Cys)	N	N	N	N	N
c.4462G>A	p.(Glu1488Lys)	N	N	N	N	0 → 62.7 gain BP at c.4462
c.4868T>G ^#^	p.(Val1623Gly)	N	N	N	N	N
c.5103A>G	p.(Lys1701=)	N	N	N	N	N
c.6011A>T ^#^	p.(Asp2004Val)	N	N	N	N	53.1 → 0 loss BP at c.6011
c.6424T>C	p.(Ser2142Pro)	N	N	N	N	N
c.6802T>C	p.(Phe2268Leu)	N	N	0 → 0.43 gain PSS at c.6805	1.96 → 3.35 (+71.1%) at c.6805	N

^#^ variants identified in the same patient; N = no significant alterations; PSS = potential splice site; BP = branch point.

**Table 4:** Clinical features of patients carrying *NOTCH2* variants and genetic alterations identified in other genes/chromosomal regions.

Patient	Variants in *NOTCH2* Gene	Variants in Other Genes	Age of Onset/ Genetic Analysis	Sex	ALGS Phenotype	Cholestasis	Pruritus	Bile Duct paucity/Age of Biopsy	Hypertransaminasemia	γ GT Elevation	Other Hepatic Features	Cardiac and Vascular Alterations	Butterfly Vertebrae	Facial Features	Eye Features	Kidney Alterations	Hypercholesterolemia	Growth	Other Clinical Conditions
1	c.665A>G–p.(Tyr222Cys)	-	4 years	F	Yes	Yes	No	NE	Yes	Yes	-	Pulmonary artery stenosis; mild bicuspid insufficiency	Yes	No	No	No	No	Normal	-
2	c.1583_1590delTTTGCCAG–p.(Val528Aspfs*2)	*JAG1* c.94T>C–p.(Ser32Pro)	1 month	F	Yes	Yes	Yes. Very Severe	Yes/2 months	Yes	Yes	Liver transplantation at 19 months	Pulmonary artery stenosis	No	Yes	No	ARF post liver transplant	Yes	SGA; poor growth before liver transplantation	Recurrent post-transplant otitis
3	c.2762A>G–p.(Gln921Arg)	-	61 years	M	No	No	No	No/61 years	Yes	No	Polymorphic hepatocytes and steatosis	-	NE	No	Hypertensive retinopathy; slight posterior vitreous detachment	Cyst of the left kidney	Yes	Normal	Metabolic syndrome; parkinsonism; bipolar disorder; apathy
4	c.3142C>T–p.(Arg1048Cys)	-	5 years	F	No	No	No	NE	Yes	No	Mild hepatomegaly and fibrosis	-	No	Mild	No	No	Yes	Normal	-
5	c.4462G>A–p.(Gln1488Lys)	-	3 years	F	No	No	No	NE	No	No	Biliary sludge	Small PFO	NE	No	No	No	No	Normal	Acute pancreatitis; thalassemia trait
6	c.4868T>G–p.(Val1623Gly); c.6011A>T–p.(Asp2004Val)	-	2 months	F	Yes	Yes	No	Modest paucity/7 months	No	Yes	Steatosis, modest neoductulation, and megasinusoids	Patency of Botallo’s duct surgically corrected; ventricular SD	No	Yes	No	No	No	Normal	Thyroiditis in euthyroidism; osteopenia
7	c.5103A>G–p.(Lys1701=)	*COG5* c.298C>T–p.(Leu100Phe) and *ALG1* c.946G>A–p.(Val316Ile)	2 months	M	Yes	Yes	No	NE	Yes	Yes	-	PFO (spontaneous resolution)	Yes	No	No	No	No	SGA	-
8	c.6007C>T–p.(Arg2003*)	-	1 month	M	Yes	Yes	Yes. Very severe	NE	Yes	Yes	Hypoplastic gallbladder	PFO; slight tricuspid insufficiency	NE	Yes	No	Slight dilation of renal calyces	Yes	SGA; poor growth	Otitis; hypogenitalism
9	c.6424T>C–p.(Ser2142Pro)	CGH array: deletion of 2q34, 9p21.1, 13q14.2 and duplication of 22q11.21	1 month	M	Yes	Yes	Yes	NE	Yes	Yes	-	Atrial SD; hypoplasia transverse sinuses	No	Yes	No	Pielectasy	No	Normal	Mild hypotonia with partial agenesis of the corpus callosum; mild psychomotor impairment
10	c.6802T>C–p.(Phe2268Leu)	-	1 month	M	Yes	Yes	No	NE	Yes	Yes	-	-	No	No	Keratokonus; maculopathy; retinal detachment	Bilateral renal hypoplasia evolved into CKD (waiting renal transplant)	No	Normal	Diabetes

F = female; M = male; NE = not evaluated; PFO = patent foramen ovale; SD = septal defect; ARF = acute renal failure; SGA = small for gestational age; CKD = chronic kidney disease; CGH = comparative genomic hybridization.

## Data Availability

Data are available from the corresponding author upon reasonable request.
